# Management of the Cord

**Published:** 1889-01

**Authors:** W. T. Richmond

**Affiliations:** Manor, Texas


### MANAGEMENT OF THE CORD.

By W. T. Richmond, M. D., Manor, Texas.

Read before the Austin District Medical Society December 20, 1888, and ordered printed in Daniel's Texas Medical Journal.

ABOUT seven years ago I was induced to consider the propriety of leaving the newly born child without a bandage and the stump of the umbilical cord without dressing. A favorable opportunity offering to put the plan into practice, I was so well pleased with the result that I have discarded the bandage and dressing, in all cases left to my discretion, where there is no special indication for them.

As soon as the child is born I place it so that its movements will not disturb the mother. I then place a ligature on the cord three or more inches from the abdomen ; a second ligature is applied about an inch from the first, and the cord divided between them. The child is then given to the nurse who is instructed to wash it in the usual manner. When the child is ready for its dress, I cut the cord again; this time about an inch and a half from the abdomen, envelop the stump in a piece of cloth, and by pressing and stripping remove all the blood, and a part of the jelly contained in it, after which I place a ligature near its extremity and have the child dressed without a bandage. An examination on the following day will show the cord shrunken and drying ; on the next day it has much the appearance of a dry scab about the size of a twenty-five cent piece. It will often fall off on the third day, leaving the umbilicus resembling a scar, from which the scab has recently fallen.

I know of no advantage the bandage is to the child. It soon becomes soiled and disarranged and is a source of annoyance. It interferes with respiration and increases the danger of hernia in paroxysms of coughing and sneezing, prevents frequent easy examinations of the stump, and renders treatment difficult, if any should be required.

DISCUSSION.

Dr. Talley endorsed, in the main, the sentiments expressed in Dr. Richmond's paper. He thought it was time for progressive physicians to begin to enquire into traditions, and correct errors wherever they may be found. He had himself, for a mumber of years, dispensed with the bandage, but he often had occasion to regret it on account of some energetic old lady entering a protest to such practice. The subject was very important, and had given him much concern. He regarded the bandage as entirely superfluous, and often actually injurious. It causes hernia by forcing the abdominal contents down against the weak ring. The largest per cent. of so called congenital hernia is produced by the bandage. By its compression colic is produced, and many other discomforts often attending the first days of childhood. He breaks up the cord, and compresses it by placing on a cloth and squeezing it. Never greases the cord but powders it, and it soon dries up like a scab. The bandage is a cruel tradition, and should be done away with. It causes dragging upon the cord, and produces pain which is often taken for colic.

Dr. Wooten thought to not use the bandage, and allow the cord to dangle and be pulled upon by the clothing, one had as well leave the child naked at once. A bandage never did harm if perfectly applied. He pressed the cord after cutting it, used absorbent cotton, and then put on the bandage to fit, and observed strict cleanliness. He had never regretted this method.

Dr. Denton admired the style of Dr. Richmond's paper. It was short and to the point. The subject of the management of the cord had been discussed, to his knowledge, for the last twenty years. He did not agree with the paper, nor with Dr. Talley, nor with any of those that had gone before advocating the no bandage and no tying plan. While he thought it was possible to chew the cord in two, as did some of the lower animals, he thought that human beings were not brutes, and that infants needed assistance such as intelligence, not instinct, could direct in the way of proper supports and cleanliness. He stuck to the bandage.

Dr. J. W. McLaughlin had no experience with the method advocated in the paper. He thought the bandage, with proper antisepsis, was quite sufficient, and having always had success he saw no reason for forsaking an old friend.

Dr. Gregg had seen cases treated by Dr. Richmond, and could testify to the success of his plan.

Dr. Monroe thought the management of the cord, as now practiced, should be much simplified. He thought the bandage was often too thick, too stiff and too broad for either comfort or efficiency. The diaper was often four fold, and large enough for half a dozen. Only the softest cloth should be used, and just enough to answer the purpose. He often found it difficult to keep old women from managing the cord themselves.

Dr. Richmond replied that he saw no objection to leaving the child naked, provided it be kept clean and warm.

Dr. Talley proceeded to discuss the subject, and finally branched off on the treatment of hernia and was called to order, and the discussion closed.

